# Age-related cognitive decline and associations with sex, education and apolipoprotein E genotype across ethnocultural groups and geographic regions: a collaborative cohort study

**DOI:** 10.1371/journal.pmed.1002261

**Published:** 2017-03-21

**Authors:** Darren M. Lipnicki, John D. Crawford, Rajib Dutta, Anbupalam Thalamuthu, Nicole A. Kochan, Gavin Andrews, M. Fernanda Lima-Costa, Erico Castro-Costa, Carol Brayne, Fiona E. Matthews, Blossom C. M. Stephan, Richard B. Lipton, Mindy J. Katz, Karen Ritchie, Jacqueline Scali, Marie-Laure Ancelin, Nikolaos Scarmeas, Mary Yannakoulia, Efthimios Dardiotis, Linda C. W. Lam, Candy H. Y. Wong, Ada W. T. Fung, Antonio Guaita, Roberta Vaccaro, Annalisa Davin, Ki Woong Kim, Ji Won Han, Tae Hui Kim, Kaarin J. Anstey, Nicolas Cherbuin, Peter Butterworth, Marcia Scazufca, Shuzo Kumagai, Sanmei Chen, Kenji Narazaki, Tze Pin Ng, Qi Gao, Simone Reppermund, Henry Brodaty, Antonio Lobo, Raúl Lopez-Anton, Javier Santabárbara, Perminder S. Sachdev, Cohort Studies of Memory in an International Consortium (COSMIC)

**Affiliations:** 1 Centre for Healthy Brain Ageing, University of New South Wales, Sydney, Australia; 2 Rene Rachou Research Institute, Oswaldo Cruz Foundation, Rio de Janeiro, Brazil; 3 Department of Public Health and Primary Care, Cambridge University, Cambridge, United Kingdom; 4 MRC Biostatistics Unit, Institute of Public Health, Cambridge, United Kingdom; 5 Institute of Health and Society, Newcastle University, Newcastle upon Tyne, United Kingdom; 6 Saul B. Korey Department of Neurology, Albert Einstein College of Medicine, Yeshiva University, New York City, New York, United States of America; 7 Department of Epidemiology and Population Health, Albert Einstein College of Medicine, Yeshiva University, New York City, New York, United States of America; 8 Inserm, U1061 Neuropsychiatry: Epidemiological and Clinical Research, La Colombière Hospital, Montpellier, France; 9 Université de Montpellier, Montpellier, France; 10 Centre for Clinical Brain Sciences, University of Edinburgh, Edinburgh, United Kingdom; 11 National and Kapodistrian University of Athens, Athens, Greece; 12 Columbia University, New York City, New York, United States of America; 13 Harokopio University, Athens, Greece; 14 University of Thessaly, Larissa, Greece; 15 Department of Psychiatry, The Chinese University of Hong Kong, Hong Kong SAR, China; 16 Department of Psychiatry, Tai Po Hospital, Hong Kong SAR, China; 17 GolgiCenci Foundation, Abbiategrasso, Milan, Italy; 18 Department of Neuropsychiatry, Seoul National University Bundang Hospital, Seongnam, Korea; 19 Department of Psychiatry, Seoul National University, College of Medicine, Seoul, Korea; 20 Department of Brain and Cognitive Science, Seoul National University College of Natural Sciences, Seoul, Korea; 21 Department of Psychiatry, Yonsei University Wonju Severance Christian Hospital, Wonju, Korea; 22 Centre for Research on Ageing, Health and Wellbeing, College of Medicine, Biology and Environment, The Australian National University, Canberra, Australia; 23 Institute of Psychiatry and LIM-23, Clinics Hospital, University of São Paulo, São Paulo, Brazil; 24 Faculty of Arts and Science, Kyushu University, Kasuga City, Japan; 25 Xiangya School of Nursing, Central South University, Changsha, China; 26 Faculty of Socio-Environmental Studies, Fukuoka Institute of Technology, Fukuoka City, Japan; 27 Gerontology Research Programme, Department of Psychological Medicine, Yong Loo Lin School of Medicine, National University of Singapore, Singapore; 28 Dementia Collaborative Research Centre, University of New South Wales, Sydney, Australia; 29 Centro de Investigación Biomédica en Red de Salud Mental (CIBERSAM), Ministry of Science and Innovation, Madrid, Spain; 30 Department of Medicine and Psychiatry, Universidad de Zaragoza, Zaragoza, Spain; 31 Centro de Investigación Biomédica en Red de Salud Mental, CIBERSAM, Spanish Ministry of Economy and Competitiveness, Madrid, Spain; 32 Department of Microbiology, Preventive Medicine and Public Health, University of Zaragoza, Spain; University of California San Francisco Memory and Aging Center, UNITED STATES

## Abstract

**Background:**

The prevalence of dementia varies around the world, potentially contributed to by international differences in rates of age-related cognitive decline. Our primary goal was to investigate how rates of age-related decline in cognitive test performance varied among international cohort studies of cognitive aging. We also determined the extent to which sex, educational attainment, and apolipoprotein E ε4 allele (APOE*4) carrier status were associated with decline.

**Methods and findings:**

We harmonized longitudinal data for 14 cohorts from 12 countries (Australia, Brazil, France, Greece, Hong Kong, Italy, Japan, Singapore, Spain, South Korea, United Kingdom, United States), for a total of 42,170 individuals aged 54–105 y (42% male), including 3.3% with dementia at baseline. The studies began between 1989 and 2011, with all but three ongoing, and each had 2–16 assessment waves (median = 3) and a follow-up duration of 2–15 y. We analyzed standardized Mini-Mental State Examination (MMSE) and memory, processing speed, language, and executive functioning test scores using linear mixed models, adjusted for sex and education, and meta-analytic techniques. Performance on all cognitive measures declined with age, with the most rapid rate of change pooled across cohorts a moderate -0.26 standard deviations per decade (SD/decade) (95% confidence interval [CI] [-0.35, -0.16], *p* < 0.001) for processing speed. Rates of decline accelerated slightly with age, with executive functioning showing the largest additional rate of decline with every further decade of age (-0.07 SD/decade, 95% CI [-0.10, -0.03], *p* = 0.002). There was a considerable degree of heterogeneity in the associations across cohorts, including a slightly faster decline (*p* = 0.021) on the MMSE for Asians (-0.20 SD/decade, 95% CI [-0.28, -0.12], *p* < 0.001) than for whites (-0.09 SD/decade, 95% CI [-0.16, -0.02], *p* = 0.009). Males declined on the MMSE at a slightly slower rate than females (difference = 0.023 SD/decade, 95% CI [0.011, 0.035], *p* < 0.001), and every additional year of education was associated with a rate of decline slightly slower for the MMSE (0.004 SD/decade less, 95% CI [0.002, 0.006], *p* = 0.001), but slightly faster for language (-0.007 SD/decade more, 95% CI [-0.011, -0.003], *p* = 0.001). APOE*4 carriers declined slightly more rapidly than non-carriers on most cognitive measures, with processing speed showing the greatest difference (-0.08 SD/decade, 95% CI [-0.15, -0.01], *p* = 0.019). The same overall pattern of results was found when analyses were repeated with baseline dementia cases excluded. We used only one test to represent cognitive domains, and though a prototypical one, we nevertheless urge caution in generalizing the results to domains rather than viewing them as test-specific associations. This study lacked cohorts from Africa, India, and mainland China.

**Conclusions:**

Cognitive performance declined with age, and more rapidly with increasing age, across samples from diverse ethnocultural groups and geographical regions. Associations varied across cohorts, suggesting that different rates of cognitive decline might contribute to the global variation in dementia prevalence. However, the many similarities and consistent associations with education and *APOE* genotype indicate a need to explore how international differences in associations with other risk factors such as genetics, cardiovascular health, and lifestyle are involved. Future studies should attempt to use multiple tests for each cognitive domain and feature populations from ethnocultural groups and geographical regions for which we lacked data.

## Abstract 2

In a collaborative cohort study, Darren Lipnicki and colleagues investigate associations between age-related cognitive decline and sex, education, and apolipoprotein E genotype across ethnocultural groups and geographic regions.

## Author summary

**Why was this study done?:**

- The prevalence of dementia varies around the world, but it is not known whether international differences in rates of cognitive decline contribute to this.
- The extent to which risk and protective factors such as sex, education, and apolipoprotein E ε4 allele (APOE*4) carrier status have different associations with dementia in different ethnocultural groups and geographic regions is also not known.

**What did the researchers do and find?:**

- We analyzed cognitive performance data from 42,170 mostly elderly individuals, provided by 14 studies of aging representing 12 countries (Australia, Brazil, France, Greece, Hong Kong, Italy, Japan, Singapore, Spain, South Korea, United Kingdom, United States).
- The Mini-Mental State Examination (MMSE) and memory, processing speed, language, and executive functioning test scores all declined with age, and rates of decline accelerated with age.
- The 14 studies showed different rates of decline, and decline in MMSE scores was faster for Asians than whites, females than males, and APOE*4 carriers than non-carriers. APOE*4 carriers also declined faster than non-carriers on test of memory, processing speed, and language.

**What do these findings mean?:**

- International differences in rates of cognitive decline might contribute to the global variation in dementia prevalence.
- Further research is needed to determine whether cardiovascular health, lifestyle, and other risk factors for dementia have different associations with cognitive decline in different ethnocultural groups and geographic regions.

## Introduction

The age-specific prevalence of dementia varies around the world, reportedly being the highest in Latin America and lowest in sub-Saharan Africa [1]. While age-specific prevalence is a good indicator of the population burden of dementia, the relative risk of dementia in different countries is better reflected in the age-specific incidence data. Unfortunately, such data are frequently lacking, especially in low- and middle-income countries. A reasonable proxy for dementia incidence is the rate of cognitive decline, with the expectation that more rapid cognitive decline will lead to higher rates of dementia in one population than another. Differences in the rates of cognitive decline may also contribute to global variation in late-life cognitive deficits less severe than dementia, such as the prevalence of mild cognitive impairment (MCI), which varies even when applying uniform diagnostic criteria [2], and performance on immediate word list recall tasks [3].

Different rates of cognitive decline have been a focus of research seeking to account for lower cognitive performance scores and more prevalent dementia among blacks than whites in the United States [4]. However, the results have been mixed, with some studies suggesting faster decline in whites [5,6] but others suggesting faster decline in blacks [7,8] or no difference between blacks and whites [7,9,10]. There are only a few studies comparing rates of late-life cognitive decline among international cohorts. One of these studies reported a similar rate of decline in immediate word list recall scores across samples from Europe, the US, China, and Mexico but a slower rate of decline in a sample from India [3]. However, not only were these findings based on one type of cognitive task, they were derived primarily from cross-sectional data and, thus, complicated by cohort effects. Another study found no difference in how Mini-Mental State Examination (MMSE) scores declined with age across six samples from four countries [11], but all of these had predominantly white populations with presumably less cultural and socioeconomic disparities than present among a broader range of international regions.

Given the current state of the research, it is not yet known whether different rates of cognitive decline contribute to the global variation in cognitive functioning and rates of dementia. Also unknown is the extent to which risk and protective factors have different associations with cognitive decline in different ethnocultural groups and geographic regions. One important factor is sex, with decline in cognitive performance found to occur more rapidly in women than men in a Chinese cohort, but not in a Danish cohort [12]. The apolipoprotein E ε4 allele (APOE*4) is an established risk factor for Alzheimer disease (AD) [13] and for the transition from MCI to AD [14]. However, the prevalence of APOE*4 among AD patients varies across geographic regions and is significantly lower in Asia than in Europe and North America [15]. Educational attainment has been considered a likely determinant of cognitive decline rates, but research has been inconclusive, with different (even opposite) effects found in different cohorts [7]. Educational attainment is also a factor that varies substantially among late-life cohorts from around the world (e.g., as shown in Sachdev et al. [2]).

The Cohort Studies of Memory in an International Consortium (COSMIC) is a collaborative effort comprising members from around the world who share data from current or past longitudinal population-based studies of cognitive aging [16]. For the current study, data were available for 14 cohorts, representing 12 countries from North and South America, Europe, Asia, and Australia. Our primary goal was to harmonize these data and compare the rates of age-related decline on various types of cognitive tests across the samples. We also aimed to investigate the extent to which sex, educational attainment, and APOE*4 carrier status were associated with decline. Knowing whether rates of cognitive decline differ across different ethnocultural groups and geographic regions will help to clarify why there is global variation in cognitive functioning and prevalence of dementia. With such a diverse overall sample, our study should also help to clarify how sex, education, and APOE*4 carrier status are associated with cognitive decline.

## Methods

### Contributing studies and participants

The total sample size of 42,170 individuals for this project was arrived at by combining the samples of all 14 COSMIC studies contributing longitudinal cohort data (listed in Table 1 with their abbreviations). In most cases the full cohort was not used, as we excluded individuals missing data for age, sex, or years of education. The samples we used varied in size from 785 to 12,630 individuals.

**Table 1:** Contributing studies.

Study	Abbreviation	Location	Start–end date	N*	Reference
Bambui Cohort Study of Aging	Bambui	Bambui, Brazil	1997–2013	1,557	Lima-Costa et al. [17]
Cognitive Function & Ageing Study	CFAS	United Kingdom†	1989–	12,630	Brayne et al. [18]
Einstein Aging Study	EAS	New York, USA	1993–	2,236	Katz et al. [19]
Etude Santé Psychologique et Traitement	ESPRIT	Montpellier, France	1999–	2,257	Ritchie et al. [20]
Hellenic Longitudinal Investigation of Aging and Diet	HELIAD	Larissa, Greece	2010–	1,228	Dardiotis et al. [21]
Hong Kong Memory and Ageing Prospective Study	HK-MAPS	Hong Kong	2005–	785	Wong et al. [22]
Invecchiamento Cerebrale in Abbiategrasso	Invece.Ab	Abbiategrasso, Italy	2010–2015	1,320	Guaita et al. [23]
Korean Longitudinal Study on Cognitive Aging and Dementia	KLOSCAD	South Korea‡	2009–2018	6,832	Kim et al. [24]
Personality and Total Health Through Life Project	PATH	Canberra, Australia	2001–2021	2,546	Anstey et al. [25]
São Paulo Ageing & Health Study	SPAH	São Paulo, Brazil	2003–2008	1,957	Scazufca et al. [26]
Sasaguri Genkimon Study	SGS	Sasaguri, Japan	2011–	2,186	Narazaki et al. [27]
Singapore Longitudinal Ageing Studies (I)	SLASI	Singapore	2003–	855	Feng et al. [28]
Sydney Memory and Ageing Study	Sydney MAS	Sydney, Australia	2005–	1,037	Sachdev et al. [29]
Zaragoza Dementia Depression Project	ZARADEMP	Zaragoza, Spain	1994–	4,744	Lobo et al. [30]

* Size of the analyzed sample, with individuals missing age, sex, or years of education data excluded from the original cohort.

† Five identical centers including Cambridgeshire, Gwynedd, Newcastle, Nottingham, and Oxford.

‡ Nationwide.

Contributing studies had various assessment schedules and follow-up durations. The number of assessment waves (including baseline) was two for six studies, three for five studies, four for two studies, and 16 for two studies (Bambui and EAS), and the maximum follow-up duration was between 2 and 10 y for all studies except Bambui and EAS (each 15 y). For CFAS, the number and type of follow-up assessments differed among the participants (see http://www.cfas.ac.uk/cfas-i/cfasistudy-design/), and we used an abridged schedule comprising baseline and two follow-up waves that captured the majority of participants (waves S0, C2/S2, CX). For each cohort and assessment wave, the number of participants assessed and the average time since baseline are shown in S1–S3 Tables.

### Ethics approval

This COSMIC project was approved by the University of New South Wales Human Research Ethics Committee (Ref: # HC12446). Each of the 14 contributing studies had previously obtained ethics approval from their respective institutional review boards, and all participants provided informed consent (see S4 Table for details). Further participant consent was not required, as de-identified health data are not considered to be protected health information under current research principles (e.g., as per the Privacy Rule proposed by the National Institutes of Health: http://privacyruleandresearch.nih.gov/research_repositories.asp).

### Measures

We obtained information on age, sex, educational attainment, and dementia status at baseline from all studies. Data for educational attainment were provided as years by all studies except ESPRIT, for which categories (e.g., higher primary, long technical or professional) had to be assigned discrete year values based on informed decisions. All but four studies also provided APOE*4 carrier status data (see the references in Table 1 for collection details), which we classified as carriers of one or two ε4 alleles versus non-carriers. Cognitive performance was assessed with scores for the MMSE [31] and for neuropsychological tests representing each of four cognitive domains: memory, language, processing speed, and executive functioning (a visuospatial domain was not included because there were not enough common relevant tests across the cohorts). All studies except EAS and SPAH administered the MMSE. However, EAS administered the Blessed Information Memory Concentration test, and a validated formula was used to convert these scores to MMSE scores [32]. Scores for neuropsychological tests from one or more of the cognitive domains were available for all studies except Bambui, SGS, and ZARADEMP. For each of the domains, we used a single test or type of test as common to all studies as possible. For memory, this was a delayed word list recall test, though the particular test varied between studies. The most commonly used memory test was the Rey Auditory Verbal Learning Test [33]. For studies without a specific memory test, we used the MMSE three-word recall sub-score. Tests were allocated to the remaining domains in a manner reflecting common practice [33–35], though we acknowledge that opinions vary and our approach may differ from how the studies previously allocated tests to domains. We allocated semantic fluency tests, typically the number of animals named in 60s [33], to language (as per Ganguli et al. [36]). Trail Making Tests A and B [33] were allocated to processing speed and executive functioning, respectively (as endorsed by others, e.g., Lim et al. [37]). The tests from each study and variations in type or nonstandard administration are detailed in S5 Table.

### Statistical analysis

The following analyses were performed separately for each cohort and each test. First, scores greater than three standard deviations (SDs) from the mean were considered outliers and excluded (the proportions of outliers and other missing scores are shown in S6 Table). Where required, a logarithmic or other transformation was applied to reduce a distribution’s absolute value of skewness from >1 to <1 before identifying outliers. Next, linear mixed models with random effects terms for intercept and age (but not age^2^) were applied to the original, untransformed data (with outliers removed) to produce estimates of the mean and SD for common values of age (75 y), education (9 y), and sex (50% female). These estimates were used to transform the raw test scores to standardized Z-scores by subtracting the estimated means from the raw scores and then dividing this difference by the estimated SD. These Z-scores were then used in analyses examining longitudinal associations with age, sex, education, and APOE*4 carrier status. Across the contributing studies, standardized scores for the different tests (or different versions or administration protocols for the same test) used to represent a domain were regarded as equivalent in that they provided a comparable metric to compare effect sizes for relationships across studies and between different types of tests.

The type of analysis employed was dependent upon whether the distribution of test scores was approximately symmetric (|skewness| <1) or more highly skewed (|skewness| >1). If approximately symmetric, linear mixed modelling was used, with fixed effects for age, age^2^, sex, education, and interactions of both sex and education with age, and with random effects for the intercept and age (but not age^2^). Age was centered at 75 y (approximately the mean age across all cohorts and waves) to reduce multicollinearity between age and age^2^. For more highly skewed distributions, we used generalized linear mixed effect modelling with the gamma distribution, featuring the same fixed and random effects as above. Note that because age was centered at 75 y, estimates of the fixed effects of age obtained from the above models (that include an age^2^ term in the equations) represent model estimates of longitudinal associations with age at 75 y. Associations with APOE*4 carrier status were also investigated by repeating the above analyses, with this variable, as well as its interaction with age, included in the model. The dominant genetic model was used, and there was no race-based stratification in comparisons of ε4 carriers and non-carriers.

Estimates of effect sizes pooled across samples were obtained by meta-analysis (using random effects models) and presented in forest plots. Heterogeneity of effect sizes among samples was evaluated with the I^2^ statistic, which is the percentage of the total variation that is due to variation between studies, rather than sampling error or chance. We report I^2^ values derived from fixed effects models that give more appropriate indications of variation across studies. We took values of I^2^ as corresponding to levels of heterogeneity that were low if less than 40%, moderate if 40%–60%, and substantial or considerable if greater than 60% (as per the Cochrane Collaboration [38]).

We repeated our analyses separately for two racial/ethnic groups, one with all individuals from cohorts predominantly comprising white participants (CFAS, ESPRIT, HELIAD, Invece.Ab, PATH, Sydney MAS, ZARADEMP) and one with all individuals from cohorts predominantly comprising Asian participants (HK-MAPS, KLOSCAD, SGS, SLASI). The statistical significance of differences in pooled corresponding cognitive measures between the two groups was obtained using the means and standard errors (SEs) of the pooled measure derived from the meta-analyses. The SE of the difference between two pooled measures (SE_diff_) was calculated as the square root of the sum of the squares of the SEs of the two pooled measures. Differences between the means greater than 1.96 times SE_diff_ were regarded as statistically significant.

Meta-analyses were also used to obtain pooled estimates of fixed effects of sex, education, and APOE*4 carrier status, as well as the interactions of these risk factors with age, and to examine how consistent these associations were across cohorts. Age was analyzed in years, but for ease of interpretation, Bs and 95% confidence intervals (CIs) are presented using age in decades. CIs were obtained as $B\pm Z_{\frac{\alpha }{2}}SE(B)$, where B is the estimate, SE(B) is the standard error of B, and $Z_{\frac{\alpha }{2}}$ is the upper 97.5% percentile point of the standard normal distribution.

In a number of study/test distributions, ceiling or floor effects had produced data spikes in which a relatively large proportion of scores were of either the minimum or maximum possible value. The most prominent reason for this was the termination of timed tests after a predetermined period and the recording of a score equal to that time. These scores were removed in order to achieve convergence or acceptable model fit (the numbers of scores removed are shown in S6 Table). We subsequently examined whether the removal of these scores affected our results by repeating the meta-analyses used to obtain pooled estimates of the fixed effects for each cognitive measure with studies featuring data spikes excluded.

Our primary analyses used data from all available individuals with sufficient information, including those identified by the contributing studies as having dementia at baseline. The inclusion of individuals with dementia at baseline meant that our evaluations of cognitive change were more likely to truly represent those of aging populations. However, as it is possible that individuals with dementia may decline at rates different from those without dementia, the analyses were repeated with cases of baseline dementia removed. The majority of studies diagnosed or classified dementia using DSM-IV criteria, with the exceptions being Bambui (an MMSE score cutoff point 13/14 appropriate for Brazilian populations with low schooling [39]), CFAS (AGECAT organicity level of O3), ESPRIT (standardized interview by a neurologist incorporating cognitive testing, with diagnoses validated by an independent panel of expert neurologists), HK-MAPS (Clinical Dementia Rating ≥1), and SGS (self-reported medical history). We note that these approaches are not harmonized or necessarily optimal for identifying dementia, including the case in which MMSE scores were used while other criteria for dementia, such as impaired functional ability, were not considered.

The Sydney team created the pooled dataset and performed the analyses. IBM SPSS Statistics 23 was used to create the dataset and identify outliers, the function Ime in the R (version 3.3.1) package mlme (https://www.r-project.org/) was used for linear mixed effects modelling, and the Penalised Quasi-Likelihood method implemented in the program glmPQL of the MASS package [40] was used for generalized linear mixed effects modelling. The meta-analyses were conducted and forest plots made using the metafor package in R [41].

## Results

### Sample description

Table 2 shows the demographic characteristics of the 14 cohorts contributing to our longitudinal analyses. All of the cohorts except one (PATH) had a greater proportion of females than males. For nine of the cohorts, the age of the youngest participant at baseline was 60 or more years (it was no less than 54 y for the remaining cohorts). The design of both Invece.Ab and PATH led to their cohorts having a much narrower age range than others. While most cohorts contained some participants with no formal schooling, the mean number of years of education varied considerably. Participants from the Brazilian cohorts (Bambui and SPAH) had the fewest years of formal education. Each cohort was essentially homogenous for race/ethnicity, except for EAS (approximately two-thirds white and one-third black), Bambui (white, black, and Brazilian indigenous native), and SPAH (mostly mixed race and white). Across the cohorts, the total number of individuals with APOE*4 data was 15,199, and 22.9% of these were APOE*4 carriers. However, the proportion of APOE*4 carriers varied across the cohorts, being lowest for the two comprising predominantly Chinese participants (HK-MAPS and SLASI). Across all cohorts, the proportion of individuals with dementia at baseline was between almost zero and 5.8% (not counting two studies that excluded individuals with dementia during recruitment: HK-MAPS and Sydney MAS). The overall proportion of individuals with dementia at baseline was 3.3%.

**Table 2:** Characteristics of the cohorts at baseline.

Study	Age, y		Sex, no. (%)		Education, y		Main race/ ethnicity	Dementia, no. (%)	APOE*4, no. (%)	
	Range	Mean (SD)	Female	Male	Range	Mean (SD)			Carrier	Missing data
Bambui	60–95	69.2 (7.3)	937 (60.2)	620 (39.8)	0–14	2.7 (3.0)	Brazilian	66 (4.2)	344 (25.0)	183 (11.8)
CFAS	64–105	75.2 (6.9)	7,565 (59.9)	5,065 (40.1)	0–34	10.0 (2.3)	White	374 (3.0)	239 (23.5)	11,611 (91.9)
EAS	63–100	78.3 (5.4)	1,383 (61.9)	853 (38.1)	0–25	13.1 (3.7)	White, Black*	126 (5.6)	227 (23.6)	1,274 (57.0)
ESPRIT	65–96	73.3 (5.7)	1,313 (58.2)	944 (41.8)	0–15	10.2 (3.8)	White	69 (3.1)	436 (19.8)	52 (2.3)
HELIAD	54–94	73.2 (5.7)	712 (58.0)	516 (42.0)	0–21	6.5 (4.2)	White	48 (3.9)	-	-
HK-MAPS	60–96	72.3 (7.2)	421 (53.6)	364 (46.4)	0–20	4.8 (4.7)	Chinese	0 (0.0)†	37 (14.1)	522 (66.5)
Invece.Ab	70–75	72.2 (1.3)	713 (54.0)	607 (46.0)	0–20	6.7 (3.4)	White	39 (3.0)	237 (18.0)	4 (0.3)
KLOSCAD	58–101	70.5 (7.1)	3,927 (57.5)	2,905 (42.5)	0–26	7.8 (5.4)	Korean	319 (4.7)	964 (24.9)	2,959 (43.3)
PATH	60–66	62.5 (1.5)	1,232 (48.4)	1,314 (51.6)	4–18	13.7 (2.8)	White	1 (0.0)	641 (27.0)	172 (6.8)
SPAH	65–102	72.3 (6.4)	1,187 (60.7)	770 (39.3)	0–19	2.4 (2.9)	Brazilian	99 (5.1)	-	-
SGS	65–96	73.6 (6.2)	1,255 (57.4)	931 (42.6)	0–23	11.0 (2.5)	Japanese	8 (0.4)	-	-
SLAS I	55–91	65.3 (7.3)	531 (62.1)	324 (37.9)	0–22	6.6 (4.6)	Chinese	20 (2.3)	134 (15.8)	6 (0.7)
Sydney MAS	70–90	78.8 (4.8)	572 (55.2)	465 (44.8)	3–24	11.6 (3.5)	White	0 (0.0)†	218 (22.6)	73 (7.0)
ZARADEMP	58–102	73.9 (9.7)	2,736 (57.7)	2,008 (42.3)	1–18	7.1 (3.8)	White	202 (4.3)	-	-
Total	54–105	72.8 (7.7)	24,484 (58.1)	17,686 (41.9)	0–34	8.8 (4.6)	White	1,371 (3.3)	3,477 (22.9)	26,971 (64.0)‡

APOE*4, apolipoprotein E ε4 allele.

* White 66.5%, black 27.6%.

† Individuals with dementia were excluded during recruitment.

‡ Includes cohorts with no APOE*4 data available or supplied.

### Rates of cognitive decline

#### Fixed effects of age and age^2^

**Fig 1:**
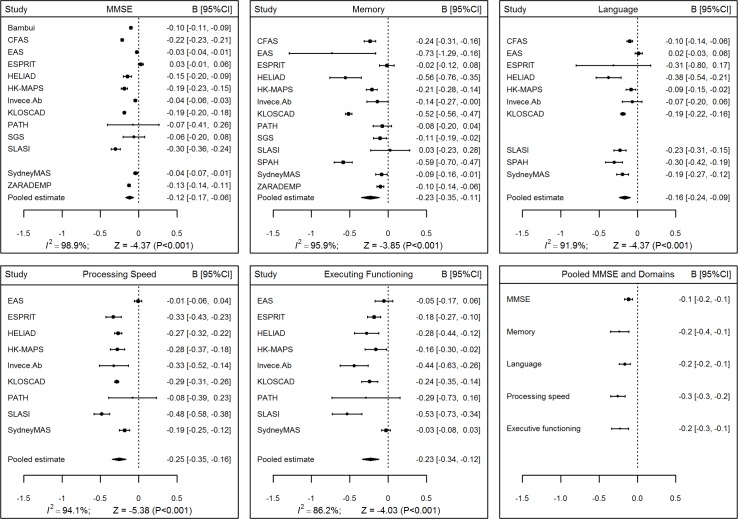
Forest plots for associations between age and Mini-Mental State Examination (MMSE) and cognitive domain scores. The *x*-axis plots change in Z-score units per decade; negative values indicate decline with age.

Across all cohorts, analyses adjusting for sex and education revealed statistically significant negative pooled associations between age and performance on the MMSE and all four cognitive domains (Fig 1, values in S7 Table). This demonstrates decline, with the rate most rapid for processing speed (-0.26 Z-score units per decade, 95% CI [-0.35, -0.16], *p* < 0.001) and least rapid for the MMSE (-0.12 Z-score units per decade, 95% CI [-0.17, -0.06], *p* < 0.001). There was a relatively high level of consistency in the direction of these associations with age but a considerable degree of variation in their size across cohorts, with high levels of heterogeneity indicated by I2 values between 86.2% and 98.9%.

Results showed small but statistically significant negative pooled associations between age^2^ and all cognitive measures, indicating decline becoming more rapid with increasing age, with the strongest associations detected for processing speed and executive functioning (S1 Fig, values in S8 Table). The associations with age^2^ exhibited high levels of heterogeneity among the cohorts for the MMSE and each of the cognitive domains, with values of I^2^ ranging from 74.0% to 96.3%.

**Fig 2:**
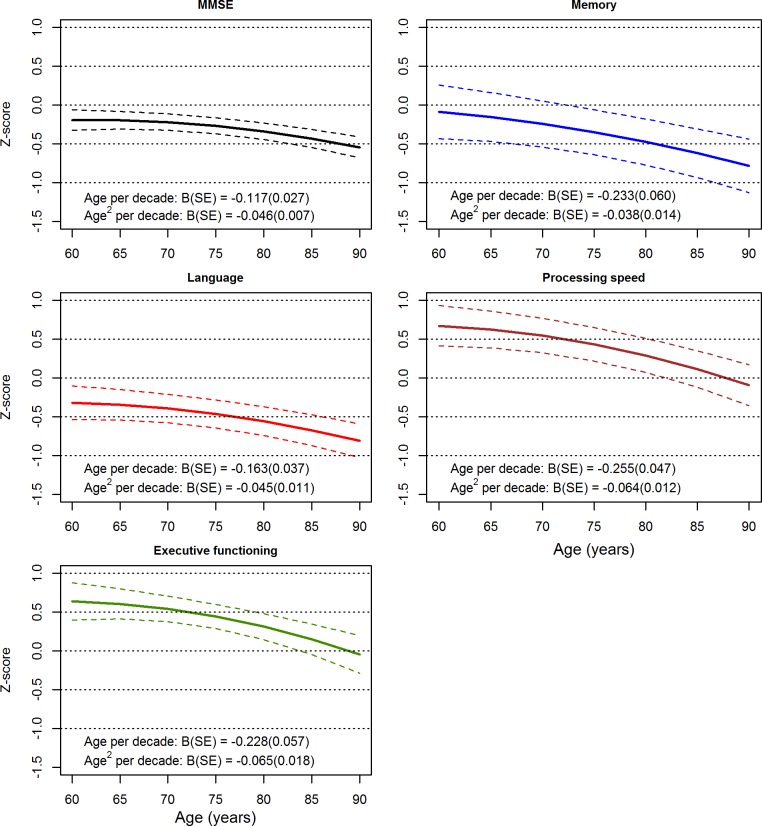
Longitudinal variation with age for the Mini-Mental State Examination (MMSE) and cognitive domains. Results were calculated from pooled estimates of the fixed effects of age and age^2^, adjusted for sex and education. Dashed lines indicate 95% confidence bands.

Fig 2 displays the longitudinal trajectories with age for the MMSE and each of the cognitive domains, calculated using the pooled estimates of the fixed effects of age and age^2^ adjusted for sex and education (as per S7 and S8 Tables), as well as 95% confidence bands calculated from the standard errors of these pooled estimates and the intercept. Each panel shows declining performance with increasing age; steepening of the slopes with age reflects the negative age^2^ fixed effects.

#### Rates of decline for white and Asian groups

**Fig 3:**
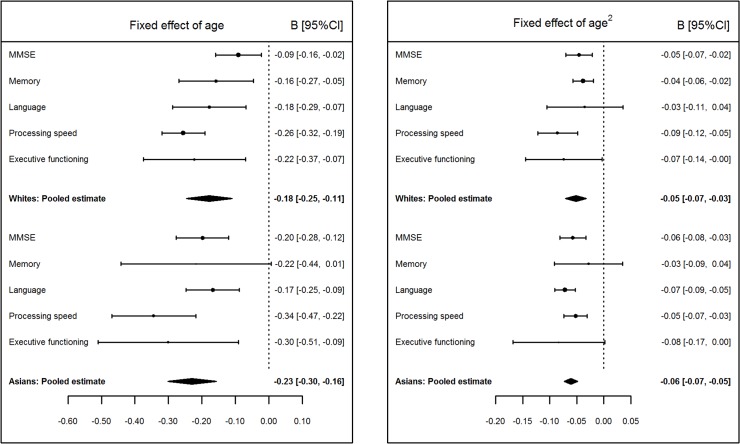
Pooled values of age and age^2^ fixed effects on Mini-Mental State Examination (MMSE) and cognitive domain scores for white and Asian groups. The *x*-axis plots change in Z-score units per decade; negative values indicate decline with age (left panel) and accelerating decline with age (right panel).

Fig 3 shows the fixed effects of age and age^2^ pooled across samples for each cognitive measure, as well as the weighted average of the pooled cognitive measures, separately for the white and Asian groups (values in S9–S12 Tables). Mirroring the finding for all cohorts analyzed as a whole, the most rapid rate of decline within each of the white and Asian groups was for processing speed. The Asian group had a slight overall tendency for higher rates of decline with age than the white group, as reflected in the small difference in the averages of pooled scores shown in Fig 3. However, across all cognitive measures and comparisons of age and age^2^ effects, the only statistically significant group difference was for the fixed effect of age on MMSE scores (B_diff_ = 0.0110, 95% CI [0.0004, 0.0211], *p* = 0.021), with a faster rate of decline in the Asian group than in the white group. Fig 3 also suggests that the difference in the strengths of the pooled age^2^ fixed effects between processing speed and language is greater for whites than for Asians.

### Associations with sex, education, and APOE*4 carrier status, as well as their interactions with age

#### Sex

Values for the pooled associations with sex, and its interaction with age, are shown for each cognitive measure in S13 and S14 Tables. The strongest association with sex was for memory (B = -0.16, 95% CI [-0.25, -0.08], *p* < 0.001), with females performing better than males. Males tended to perform better than females on all other cognitive measures, but not statistically significantly so for any measure. The interactions of sex with age were positive for all cognitive measures except processing speed, which was near zero and nonsignificant (*p* = 0.795), indicating a trend toward slightly slower decline with age for males than for females. However, this slightly slower decline for males was only statistically significant for the MMSE (B = 0.023, 95% CI [0.011, 0.035], *p* < 0.001).

#### Education

The values for the pooled associations between education and each of the cognitive measures are shown in S15 Table. Having more years of education was associated with significantly better performance on all cognitive measures. The strongest effect was for language, which exhibited an almost 0.05 Z-score unit increase for every additional year of education (B = 0.049, 95% CI [0.032, 0.066], *p* < 0.001), and the lowest for the MMSE (B = 0.011, 95% CI [0.007, 0.016], *p* < 0.001). Statistically significant interactions between education and age were found for the MMSE (B = 0.004, 95% CI [0.002, 0.006], *p* = 0.001) and language (B = -0.007, 95% CI [-0.011, -0.003], *p* < 0.001), with more years of education associated with a slower decline in MMSE scores but a faster decline in language performance (S16 Table).

#### APOE*4 carrier status

The values for the pooled associations between APOE*4 carrier status and each of the cognitive measures are shown in S17 Table. The strongest associations were with memory (B = -0.055, 95% CI [-0.075, -0.035], *p* < 0.001) and processing speed (B = -0.043, 95% CI [-0.060, -0.025], *p* < 0.001), for each of which the performance of APOE*4 carriers was around 0.05 Z-score units below that of noncarriers. APOE*4 carriers also performed significantly lower than noncarriers on the MMSE, but the negative associations with language and executive functioning were not statistically significant. There were statistically significant interactions between APOE*4 carrier status and age for all cognitive measures except executive functioning (S18 Table). The strongest interaction was for processing speed (B = -0.08, 95% CI [-0.15, -0.01], *p* = 0.019), but this was not significantly different from the interaction with any of the other measures. For all significant associations, the negative value for B indicates greater decline in cognition with age for APOE*4 carriers than for noncarriers. The results for both APOE*4 carrier status and APOE*4 carrier status by age interactions exhibited extremely low heterogeneity among the cohorts for memory and language (0.0% < I^2^ < 0.5%).

#### Repeat analyses

In order to achieve model convergence or adequate fit, our initial analyses excluded scores that formed data spikes, which were groups of scores reflecting ceiling or floor effects most prominently associated with the termination of timed tests at a predetermined time. We repeated our meta-analyses with studies featuring data spikes excluded and observed only one change to the original pooled values: the emergence of significantly lower executive functioning performance for APOE*4 carries than for noncarriers (B = -0.22, 95% CI [-0.42, -0.03], *p* = 0.025). We also performed a separate round of repeat analyses with cases of dementia at baseline removed (the pooled values of estimated fixed effects are shown in the last rows of S7–S18 Tables). While the overall pattern of results remained similar to that originally found, there were a small number of changes in the results when comparing whites and Asians and when investigating associations with sex, education, and APOE*4 carrier status. However, as can be seen in S7–S18 Tables, in most instances this involved only a small change in *p*-value and no substantial change in effect size.

## Discussion

We used individual participant-level data provided by members of the COSMIC collaboration to investigate rates of cognitive decline in 14 longitudinal population-based studies of cognitive aging, representing 12 countries and 5 continents. We also investigated the extent to which sex, education, and APOE*4 carrier status were associated with cognitive performance and decline across these diverse ethnocultural groups and geographic regions. Our findings were minimally affected when repeating our analyses with cases of baseline dementia removed, probably in large part because the overall proportion of these cases was low (3.3%).

### Main findings

The cognitive measures analyzed in this study were the MMSE and tests representing four cognitive domains: memory, language, processing speed, and executive functioning. For all of these measures and across all cohorts, we found performance to not only decline substantially with age, but to decline more rapidly with increasing age. Processing speed exhibited the strongest decline with age, and the MMSE exhibited the weakest. The rate of age-related change in processing speed was almost -0.25 Z-score units per decade, which was not too dissimilar to the rates of decline for memory and executive functioning but twice the rate of decline we found for the MMSE. This is consistent with previous reports of age-related associations being stronger for processing speed, intermediate for memory, and weaker for language [42]. Processing speed being the cognitive measure most strongly associated with age could be seen as supporting the processing speed theory of cognitive aging [43]. However, it was not our aim to investigate this, and further analyses examining the extent to which change in performance on other domains is driven by changes in processing speed would be required to test this idea. The slowest rate of decline being found for the MMSE could stem from this measure being insensitive to changes at high levels of cognition [44].

Rates of cognitive decline and changes in the rates of decline with age exhibited a degree of heterogeneity across the cohorts. The direction of associations with age was highly consistent across cohorts for all cognitive measures (with no instance of significant improvement rather than decline), but the strength differed. These differences remained when cases of baseline dementia were excluded, suggesting that they could lead to international differences in rates of incident dementia, and thereby contribute to the global variation in prevalence of dementia [1]. Future COSMIC projects will aim to harmonize data on incident dementia across the cohorts and match these to rates of cognitive decline and prevalence of dementia.

### Race/ethnicity

Despite the differences seen across all cohorts, our initial results indicated only one significant difference in rates of decline or change in rates of decline between groups of cohorts classified as white or Asian: a slightly faster decline with age on the MMSE in the Asian group. There also seemed to be a group difference in the strengths of the pooled age^2^ fixed effects between processing speed and language, which was greater for whites than for Asians. Analyses with cases of baseline dementia removed showed some additional age^2^ effects within each group, amplifying the differences between whites and Asians. Further research is needed to determine the reliability of these differences and their implications.

### Sex

Across our cohorts, females generally performed better than males on verbal memory tests, although the difference was not large. Previously reported differences in late-life memory performance between men and women have varied depending on where the samples were from. Reports of better memory in women have come from developed nations, including the UK [45], US [46], and Denmark [12]. Conversely, women have shown poorer memory performance than men in samples from developing nations or where women have historically not been afforded the same educational opportunities as men, including India [47] and China [48]. Nevertheless, it should be noted that our findings were relatively consistent across the diverse range of cohorts investigated, including some that may be from developing nations. Better verbal memory performance in women than men could arise via an effect of estrogen [49] or sex-specific cognitive reserve [50]. Our initial finding of faster decline in MMSE scores for females than for males is ostensibly consistent with reports that women exhibit both a steeper decline in general cognition with increasing age [51] and a greater prevalence of AD [52]. However, there was only a trend for this association (*p* = 0.089) after excluding baseline dementia cases from our analyses. Future COSMIC projects will use harmonized incidence of dementia data to more fully examine sex differences in cognitive decline.

### Education

Previous research has consistently found higher levels of educational attainment to be associated with better late-life cognitive functioning [7,53,54], but associations between education and rates of cognitive decline are mixed [7]. Our finding that more years of education was associated with better performance on all cognitive measures is consistent with this. Also consistent are declines with age that were slower for the MMSE but faster for language, though the reasons for the mixed directions of these associations are unclear.

### APOE*4

Compared to noncarriers, APOE*4 carriers performed worse on memory, processing speed, and the MMSE (as well as on executive functioning in analyses with studies featuring data spikes excluded). APOE*4 carriers also exhibited greater rates of decline than noncarriers for all measures except executive functioning. Such findings are not unexpected given that APOE*4 is a risk factor for AD [13] and for the transition from MCI to AD [14]. With cases of baseline dementia excluded from our analyses, APOE*4 carriers continued to show significantly poorer performance only for memory, which fits with a recent meta-analysis finding memory to be the cognitive measure most strongly affected in APOE*4 carriers with no diagnosed cognitive impairment [55]. The reasons for extremely low heterogeneity among the cohorts for associations with APOE*4 carrier status and APOE*4 carrier status by age interactions on memory and language are unclear. We note that the differences in APOE*4 carrier prevalence across our cohorts are generally consistent with previously reported racial/ethnic differences [56], particularly the relatively low prevalence for the Chinese (HK-MAPS and SLASI) and Italian (Invece.Ab) cohorts.

### Strengths and limitations

Strengths of our study include the large number of independent cohorts from diverse geographical, ethnic, and sociocultural groups and the use of the same or very similar cognitive tests by these studies. Even with analyses based on standardized scores, we expect that the use of common tests helped to minimize heterogeneity across the studies within each of the cognitive domains investigated. Nevertheless, with only one test being used to represent cognitive domains, we caution against generalizing our results to domains rather than viewing them as test-specific associations, although it is noteworthy that the tests used were prototypical of their domains, and that for the memory domain a variety of verbal memory tests were used across the cohorts. We also note that the MMSE has been criticized as psychometrically unsound for assessing cognitive change in healthy older adults [57] and prone to practice effects [58]. Indeed, with the same cognitive tests used repeatedly in all assessment waves, it is possible we underestimated age-related change because of practice effects. Being reportedly stronger in younger adults [59], practice effects could partially explain increasing rates of decline with increasing age. Other limitations include the cohorts differing in size, number of assessment waves, and overall follow-up duration. Despite all being population-based, the use of particular strategies for recruitment and regional specificity may mean that the cohorts are not necessarily representative of the countries or entire populations they were from. Our study did not have data on chronic degenerative diseases or cardiovascular and lifestyle-related factors commonly associated with aging. These factors could have independent associations with cognitive decline, and not controlling for them may lead to overestimating the strength of associations between age and cognitive decline. Limitations also come with having to harmonize some data from among a heterogeneous group of studies. For example, the use of different memory tests by the studies entailed differences in the range of possible scores, which, despite harmonization, potentially influenced the variability within studies and, thus, also potentially influenced our findings of differences between studies.

## Conclusion

In conclusion, we found that cognitive performance consistently declined with age, and more rapidly with increasing age, across cohorts from a diverse range of ethnocultural groups and geographical regions. Similar patterns of results were found for analyses that either included or excluded individuals with dementia at baseline. The strengths of the observed associations varied across the cohorts, and there were also some small differences between groups of cohorts classified as white or Asian. This suggests that different rates of cognitive decline might contribute, via different rates of incident dementia, to the global variation in dementia prevalence. Given the diversity of cohorts and our large overall sample size (more than 42,000 individuals), the associations with sex, education, and *APOE* genotype we found should help to clarify the contributions of these factors to cognitive ageing on a global scale. We intend for future research with COSMIC cohorts to explore how risk factors not investigated in the current study, including other genetic, epigenetic, cardiovascular, and lifestyle-related factors, contribute to cognitive decline and neurocognitive disorders, and to determine the extent to which their associations vary internationally. We also aim to feature populations from ethnocultural groups and geographical regions for which the current study lacked data, including Africa, India, and mainland China. This will provide important information for developing efficacious interventions to prevent or minimize cognitive impairment and dementia in the rapidly aging population worldwide.

## Supporting information

S1 STROBE Checklist STROBE checklist for cohort studies. (DOC)

S1 Fig Forest plots for the quadratic effects of age on Mini-Mental State Examination (MMSE) and cognitive domain scores. Z- and *p*-values are for the statistical tests of significance of the pooled values.
(TIF)

S1 Table Number of assessment waves, time since baseline, and number of individuals assessed with the MMSE for baseline and each follow-up wave. (DOCX)

S2 Table Time (y) since baseline and number of individuals assessed with the Mini-Mental State Examination for the 16 annual Bambui study assessment waves. (DOCX)

S3 Table Time (y, mean ± SD, and range) since baseline and number of individuals assessed with the Blessed Information Memory Concentration test for each of the 16 EAS assessment waves. (DOCX)

S4 Table Ethics approvals for the individual contributing studies. (DOCX)

S5 Table Tests from each contributing study used to represent the cognitive domains investigated. (DOCX)

S6 Table Numbers and percentages of missing and deleted test scores. (DOCX)

S7 Table Meta-analyses of the fixed effects of age on MMSE and cognitive domain scores. (DOCX)

S8 Table Meta-analyses of the fixed effects of age^2^ on MMSE and cognitive domain scores. (DOCX)

S9 Table For predominantly white studies, meta-analyses of the fixed effects of age on MMSE and cognitive domain scores. (DOCX)

S10 Table For predominantly white studies, meta-analyses of the fixed effects of age^2^ on MMSE and cognitive domain scores. (DOCX)

S11 Table For predominantly Asian studies, meta-analyses of the fixed effects of age on MMSE and cognitive domain scores. (DOCX)

S12 Table For predominantly Asian studies, meta-analyses of the fixed effects of age^2^ on MMSE and cognitive domain scores. (DOCX)

S13 Table Meta-analyses of the fixed effects of sex on MMSE and cognitive domain scores. (DOCX)

S14 Table Meta-analyses of sex by age interactions on MMSE and cognitive domain scores. (DOCX)

S15 Table Meta-analyses of the fixed effects of education on MMSE and cognitive domain scores. (DOCX)

S16 Table Meta-analyses of education by age interactions on MMSE and cognitive domain scores. (DOCX)

S17 Table Meta-analyses of the fixed effects of APOE*4 carrier status on MMSE and cognitive domain scores. (DOCX)

S18 Table Meta-analyses of APOE*4 carrier status by age interactions on MMSE and cognitive domain scores. (DOCX)

S1 Text Analysis plan for COSMIC paper on age-related cognitive decline in diverse international regions. (DOCX)

## Abbreviations

AD: Alzheimer disease
*APOE*4*: apolipoprotein E ε4 allele
CI: confidence interval
COSMIC: Cohort Studies of Memory in an International Consortium
MCI: mild cognitive impairment
MMSE: Mini-Mental State Examination
SD: standard deviation
SE: standard error
