# Supplementary material for: Age-related cognitive decline and associations with sex, education and apolipoprotein E genotype across ethnocultural groups and geographic regions: a collaborative cohort study
Source: PLoS Med. 2017 Mar 21;14(3):e1002261. doi: 10.1371/journal.pmed.1002261 (PMC5360220; doi:10.1371/journal.pmed.1002261)
Supplement: S1 STROBE Checklist — (DOC) [file pmed.1002261.s001.doc]

STROBE Statement—Checklist of items that should be included in reports of ***cohort studies***

|  | Item No | Recommendation |
| --- | --- | --- |
| **Title and abstract** | 1 | (*a*) Indicate the study’s design with a commonly used term in the title or the abstract |
|  |  | The title includes the term *cohort study.* |
|  |  | (*b*) Provide in the abstract an informative and balanced summary of what was done and what was found  The Methods and Findings section of the Abstract describes what was done and gives a balanced summary of what was found, including both easy to interpret results and those not necessarily expected and more difficult to understand, e.g., *every additional year of education was associated with a rate of decline slightly slower for the MMSE (0.004 SD/decade less [SE=0.001, p=0.001]), but slightly faster for language (0.007 SD/decade more [SE=0.001, p=0.001]).* |
| Introduction | | |
| Background/rationale | 2 | Explain the scientific background and rationale for the investigation being reported  Following an account of relevant prior research we summarize the background and rationale for our study with the following: *Given the current state of the research, it is not yet known whether different rates of cognitive decline contribute to the global variation in cognitive functioning and rates of dementia. Also unknown is the extent to which risk and protective factors have different associations with cognitive decline in different ethno-cultural groups and geographic regions.* |
| Objectives | 3 | State specific objectives, including any prespecified hypotheses  The specific objectives of our study are stated as: *Our primary goal was to harmonize these data and compare the rates of age-related decline on various types of cognitive tests across the samples. We also aimed to investigate the extent to which sex, educational attainment and APOE*4 carrier status were associated with decline.* |
| Methods | | |
| Study design | 4 | Present key elements of study design early in the paper  Key elements of study design are stated in the first sentence of the Methods: ...*by combining the samples of all 14 COSMIC studies contributing longitudinal cohort data.* |
| Setting | 5 | Describe the setting, locations, and relevant dates, including periods of recruitment, exposure, follow-up, and data collection  The salient elements of setting are detailed in *Table 1. Contributing studies*, which includes references to all 14 studies in which complete details unable to be accommodated in this paper can be found. |
| Participants | 6 | (*a*) Give the eligibility criteria, and the sources and methods of selection of participants. Describe methods of follow-up  The eligibility criteria for our study were that individuals from the 14 separate contributing study data sets were not *missing data for any of age, sex or years of education.* References to the protocols of all 14 contributing studies are included in *Table 1. Contributing studies* if study-specific details are needed. The methods of follow-up are described as *Contributing studies had various assessment schedules and follow-up durations. The number of assessment waves (including baseline) was two for six studies, three for five studies, four for two studies, and 16 for two studies (Bambui and EAS), and the maximum follow-up duration was between two and ten years for all studies except Bambui and EAS (each 15 years). For CFAS, the number and type of follow-up assessments differed among the participants (see http://www.cfas.ac.uk/cfas-i/cfasistudy-design/), and we used an abridged schedule comprising baseline and two follow-up waves that captured the majority of participants (waves S0, C2/S2, CX). For each cohort and assessment wave, the number of participants assessed and the average time since baseline are shown in Tables S1–S3.* |
|  |  | (*b*) For matched studies, give matching criteria and number of exposed and unexposed  This was not a matched study. |
| Variables | 7 | Clearly define all outcomes, exposures, predictors, potential confounders, and effect modifiers. Give diagnostic criteria, if applicable  All variables are clearly defined, e.g., *Data for educational attainment was provided as years by all studies except ESPRIT, for which categories (e.g., higher primary, long technical or professional) had to be assigned discrete year values based on informed decisions. All but four studies also provided APOE*4 carrier status data (see the references in Table 1 for collection details), which we classified as carriers of one or two ε4 alleles versus non-carriers.*  The diagnostic criteria used by the studies for dementia are given: *The majority of studies diagnosed or classified dementia using DSM-IV criteria, with the exceptions being Bambui (an MMSE score cut-off point 13/14 appropriate for Brazilian populations with low schooling {Castro-Costa, 2008}), CFAS (AGECAT organicity level of O3), ESPRIT (standardized interview by a neurologist incorporating cognitive testing, with diagnoses validated by an independent panel of expert neurologists), HK-MAPS (Clinical Dementia Rating ≥1), and SGS (self-reported medical history).* |
| Data sources/ measurement | 8* | For each variable of interest, give sources of data and details of methods of assessment (measurement). Describe comparability of assessment methods if there is more than one group  References to the protocols of all 14 contributing studies are included in *Table 1. Contributing studies* if study-specific sources of data and details of methods of assessment are of interest to the reader. Variation in assessment methods across the different groups is discussed, as is the comparability of measures following our harmonisation procedures. For example: *For each of the domains, we used a single test or type of test as common to all studies as possible. For memory this was a delayed word list recall test, though the particular test varied between studies. The most commonly used memory test was the Rey Auditory Verbal Learning Test , and for studies without a specific memory test we used the MMSE three-word recall sub-score* and *All studies except for EAS and SPAH administered the MMSE. However, EAS administered the Blessed Information Memory Concentration test, and a validated formula was used to convert these scores to MMSE scores .* |
| Bias | 9 | Describe any efforts to address potential sources of bias  We aimed to have the proportions of different cognitive performance levels within each study represent the relevant population as accurately as possible, by including only population-based studies (as opposed to clinic-based), and by performing sensitivity analyses to test whether participants with dementia at baseline affected the results (the proportion of such participants varied across studies in part due to different recruitment protocols). |
| Study size | 10 | Explain how the study size was arrived at  *The total sample size of 42,170 for this project was arrived at by combining the samples of all 14 COSMIC studies contributing longitudinal cohort data (listed in Table 1 with their abbreviations).* |
| Quantitative variables | 11 | Explain how quantitative variables were handled in the analyses. If applicable, describe which groupings were chosen and why  The handling of quantitative variables is fully detailed in the Statistical analysis section of the Methods, which begins *First, scores greater than 3 standard deviations (SDs) from the mean were considered outliers and excluded. Where required, a logarithmic or other transformation was applied to reduce a distribution’s absolute value of skewness from >1 to <1 before identifying outliers. Next, linear mixed models with random effects terms for intercept and age (but not age^2^) were applied to the original, untransformed data (with outliers removed) to produce estimates of the mean and SD for common values of age (75 years), education (9 years) and sex (50% female).* |
| Statistical methods | 12 | (*a*) Describe all statistical methods, including those used to control for confounding  All statistical methods are described, e.g., *The type of analysis employed was dependent upon whether the distribution of test scores was approximately symmetric (|skewness| <1) or more highly skewed (|skewness| >1). If approximately symmetric, linear mixed modelling was used, with fixed effects for age, age^2^, sex, education and interactions of both sex and education with age, and with random effects for the intercept and age (but not age^2^). Age was centred at 75 years (approximately the mean age across all cohorts and waves) to reduce multicollinearity between age and age^2^. For more highly skewed distributions, we used generalized linear mixed effect modelling with the gamma distribution, featuring the same fixed and random effects as above.* |
|  |  | (*b*) Describe any methods used to examine subgroups and interactions  Subgroups were analysed, e.g., *We repeated our analyses separately for two racial/ethnic groups, one with all individuals from cohorts predominantly comprising white participants (CFAS, ESPRIT, HELIAD, Invece.Ab, PATH, Sydney MAS, ZARADEMP), and one with all individuals from cohorts predominantly comprising Asian participants (HK-MAPS, KLOSCAD, SGS, SLASI). The statistical significance of differences in pooled corresponding cognitive measures between the two groups was obtained using the means and standard errors (SEs) of the pooled measure derived from the meta-analyses. The SE of the difference between two pooled measures (SE_diff_) was calculated as the square-root of the sum of the squares of the SEs of the two pooled measures. Differences between the means greater than 1.96 times SE_diff_ were regarded as statistically significant.*  Methods for examining interactions, e.g., *linear mixed modelling was used, with fixed effects for age, age^2^, sex, education and interactions of both sex and education with age.* |
|  |  | (*c*) Explain how missing data were addressed  Our analyses used linear mixed models, which are able to account for missing data in longitudinal data sets. |
|  |  | (*d*) If applicable, explain how loss to follow-up was addressed  By using linear mixed models. |
|  |  | (*e*) Describe any sensitivity analyses  We repeated our analyses with cases of baseline dementia excluded, to determine whether such individuals (the proportion of which differed across studies) influenced the observed rates of cognitive decline. |
| Results | | |
| Participants | 13* | (a) Report numbers of individuals at each stage of study—eg numbers potentially eligible, examined for eligibility, confirmed eligible, included in the study, completing follow-up, and analysed  This is detailed across 3 supplementary tables (S1-S3), e.g., *Table S1.**Number of assessment waves, time since baseline (yrs, mean±SD and range), and number of individuals assessed with the MMSE for baseline and each follow-up wave.* |
|  |  | (b) Give reasons for non-participation at each stage  With 14 different contributing studies and varying numbers of assessment waves, this level of information is not appropriate here, though can be found via the references for the studies listed in Table 1. |
|  |  | (c) Consider use of a flow diagram  Fourteen different contributing studies and varying numbers of assessment waves preclude the use of a manageable flow diagram. |
| Descriptive data | 14* | (a) Give characteristics of study participants (eg demographic, clinical, social) and information on exposures and potential confounders  Detailed in *Table 2.**Characteristics of the cohorts at baseline* and described in the text, e.g., *Across the cohorts, the total number of individuals with APOE*4 data was 15199, and 22.9% of these were APOE*4 carriers. However, the proportion of APOE*4 carriers varied across the cohorts, being lowest for the two comprising predominantly Chinese participants (HK-MAPS and SLASI)*. |
|  |  | (b) Indicate number of participants with missing data for each variable of interest  In Table S6 we indicate the number and percentage of missing data for each cognitive measure, separately for each contributing study as a total across all waves (i.e., the denominator = number of participants at baseline x number of assessment waves). |
|  |  | (c) Summarise follow-up time (eg, average and total amount)  The follow-up time for each contributing study is summarised in Tables S1-S3. |
| Outcome data | 15* | Report numbers of outcome events or summary measures over time  Summary measures over time are shown as changes in cognitive performance on all cognitive tests with age in *Fig 2. Longitudinal variation with age for the Mini-Mental State Examination (MMSE) and cognitive domains.* |
| Main results | 16 | (*a*) Give unadjusted estimates and, if applicable, confounder-adjusted estimates and their precision (eg, 95% confidence interval). Make clear which confounders were adjusted for and why they were included  By convention, neuropsychological or cognitive data are adjusted for age as a significant demographic influence on test performance, and it is well established that education and sex also have considerable effects on estimates. The demographic effects of age, education and sex vary between cognitive domains and test measures. Hence, our estimates were adjusted for all three factors. It is not meaningful to present unadjusted estimates of cognition. References for this are:  Kaufman, A. S., Kaufman, J. C., Liu, X. & Johnson, C. K. How do educational attainment and gender relate to fluid intelligence, crystallized intelligence, and academic skills at ages 22-90 years? *Arch Clin Neuropsychol* 24, 153-163 (2009).  Taylor, M. & Heaton, R. Sensitivity and specificity of WAIS-III/WMS-III demographically corrected factor scores in neuropsychological assessment. *J Int Neuropsychol Soc* 7, 867-874 (2001). |
|  |  | (*b*) Report category boundaries when continuous variables were categorized  No continuous variables were categorized. |
|  |  | (*c*) If relevant, consider translating estimates of relative risk into absolute risk for a meaningful time period  We calculated rates of cognitive decline, and not risk of any event. |
| Other analyses | 17 | Report other analyses done—eg analyses of subgroups and interactions, and sensitivity analyses  Other analyses include subgroups and interactions, e.g., *Values for the pooled associations with sex, and its interaction with age, are shown for each cognitive measure in Tables S9 and S10. The strongest association with sex was for memory (B =-0.16, SE=0.045, p<0.001), with females performing better than males. Males tended to perform better than females on all other cognitive measures, but not statistically significantly so for any measure. The interactions of sex with age were positive for all cognitive measures except processing speed, which was near zero and non-significant (p=0.795), indicating a trend toward slightly slower decline with age for males than for females. However, this slightly slower decline for males was only statistically significant for the MMSE (B=0.023, SE=0.006, p<0.001).*  Sensitivity analyses: *We also performed a separate round of repeat analyses with cases of dementia at baseline removed (the pooled values of estimated fixed effects are shown in the last rows of Tables S7a to S14). While the overall pattern of results remained similar to that originally found, there were a small number of changes in the results when comparing whites and Asians, and when investigating associations with sex, education and APOE*4 carrier status. However, as can be seen in Tables S7a to S14, in most instances this involved only a small change in p-value and no substantial change in effect size.* |
| Discussion | | |
| Key results | 18 | Summarise key results with reference to study objectives  We summarise key results under subheadings linked to study objectives, including *Main Findings, Race/Ethnicity, Sex, Education and APOE*4,* e.g., *For all of these measures, and across all cohorts, we found performance to not only decline with age, but to decline more rapidly with increasing age,* and *Compared to non-carriers, APOE*4 carriers performed significantly worse on memory, processing speed and the MMSE, and exhibited greater rates of decline for all measures except executive functioning.* |
| Limitations | 19 | Discuss limitations of the study, taking into account sources of potential bias or imprecision. Discuss both direction and magnitude of any potential bias  Examples: *Limitations also come with having to harmonise some data from among a heterogeneous group of studies. For example, the use of different memory tests by the studies entailed differences in the range of possible scores, which despite harmonization potentially influenced the variability within studies, and thus also potentially influenced our findings of differences between studies*, and *with the same cognitive tests used repeatedly in all assessment waves, it is possible we under-estimated age-related change because of practice effects.* |
| Interpretation | 20 | Give a cautious overall interpretation of results considering objectives, limitations, multiplicity of analyses, results from similar studies, and other relevant evidence  Examples: *Previous research has consistently found higher levels of educational attainment to be associated with better late-life cognitive functioning , but associations between education and rates of cognitive decline to be mixed . Our finding that greater years of education were associated with better performance on all cognitive measures is consistent with this. Also consistent are declines with age that were slower for the MMSE but faster for language, though the reasons for the mixed directions of these associations are unclear*, and *Our initial finding of faster decline in MMSE scores for females than for males is ostensibly consistent with reports that women exhibit both a steeper decline in general cognition with increasing age and a greater prevalence of AD . However, there was only a trend for this association (p=0.089) after excluding baseline dementia cases from our analyses.* |
| Generalisability | 21 | Discuss the generalisability (external validity) of the study results  Examples: *Nevertheless, with only one test used to represent cognitive domains we caution against generalising our results to domains, rather than viewing them as test-specific associations*, and *Despite all being population-based, the use of particular strategies for recruitment and regional specificity may mean that the cohorts are not necessarily representative of the countries or entire populations they were from.* |
| Other information | | |
| Funding | 22 | Give the source of funding and the role of the funders for the present study and, if applicable, for the original study on which the present article is based  The following is included in the paper: *National Health and Medical Research Council of Australia Program Grant (ID 568969; PSS). For the contributing studies: The Brazilian Ministry of Health and Ministry of Science and Technology (MFLC, ECC); Major awards from the UK Medical Research Council and the Department of Health (CB, FEM, BCMS); National Institute on Health/National Institute on Aging grants (5P01 AG003949, 1R03 AG045474; RBL, MJK); Novartis (KR, JS, MLA); Alzheimer’s Association (IIRG-09-133014), ESPA-EU program Excellence Grant (ARISTEIA), which is co-funded by the European Social Fund and Greek National resources (189 10276/8/9/2011), and Ministry for Health and Social Solidarity, Greece (ΔΥ2β/οικ.51657/14.4.2009; NS, MY, ED); Mr. Lai Seung Hung & Mrs. Lai Chan Pui Ngong Dementia in Hong Kong Research Fund, and an educational fund from Eisai (LCWL, CHYW, AWTF); Fondazione Golgi Cenci and Federazione Alzheimer Italia (AG, RV, AD); Korean Health Technology R&D Project, Ministry of Health and Welfare, Republic of Korea [Grant No. HI09C1379 (A092077); KWK, JWH, THK]; National Health and Medical Research Council of Australia (Grants 973302, 179805, 157125 and 1002160; KJA, NC, PB); Welcome Trust (grant code GR066133MA) and FAPESP-Brazil (grant code 2004/12694-8; MS); Health and Labour Sciences Research Grant from the Ministry of Health, Labour and Welfare of Japan (H25-Ninchisho-Ippan-004) and a research grant from Sasaguri town, Fukuoka, Japan (SK, SC, KN); Research grants (No. 03/ 121/17/214 and No. 08/1/21/19/567) from the Biomedical Research Council, Agency for Science, Technology and Research (A_STAR) in Singapore (TPN, QG); National Health & Medical Research Council of Australia Program Grant (ID 350833; PSS, DML, NAK, JDC, AT, GA, SR, HB); Fondo de Investigación Sanitaria, Instituto de Salud Carlos III, Spanish Ministry of Health, Madrid, Spain (Grants 94/ 1562, 97/1321E, 98/0103, 01/0255, 03/0815, 06/0617, and G03/128) and Pfizer Foundation, Madrid (AL, RLA, JS). The funders had no role in study design, data collection and analysis, decision to publish, or preparation of the manuscript.* |

*Give information separately for exposed and unexposed groups.

**Note:** An Explanation and Elaboration article discusses each checklist item and gives methodological background and published examples of transparent reporting. The STROBE checklist is best used in conjunction with this article (freely available on the Web sites of PLoS Medicine at http://www.plosmedicine.org/, Annals of Internal Medicine at http://www.annals.org/, and Epidemiology at http://www.epidem.com/). Information on the STROBE Initiative is available at http://www.strobe-statement.org.
