# Supplementary figures and images for: Age-related cognitive decline and associations with sex, education and apolipoprotein E genotype across ethnocultural groups and geographic regions: a collaborative cohort study
Source: PLoS Med. 2017 Mar 21;14(3):e1002261. doi: 10.1371/journal.pmed.1002261 (PMC5360220; doi:10.1371/journal.pmed.1002261)

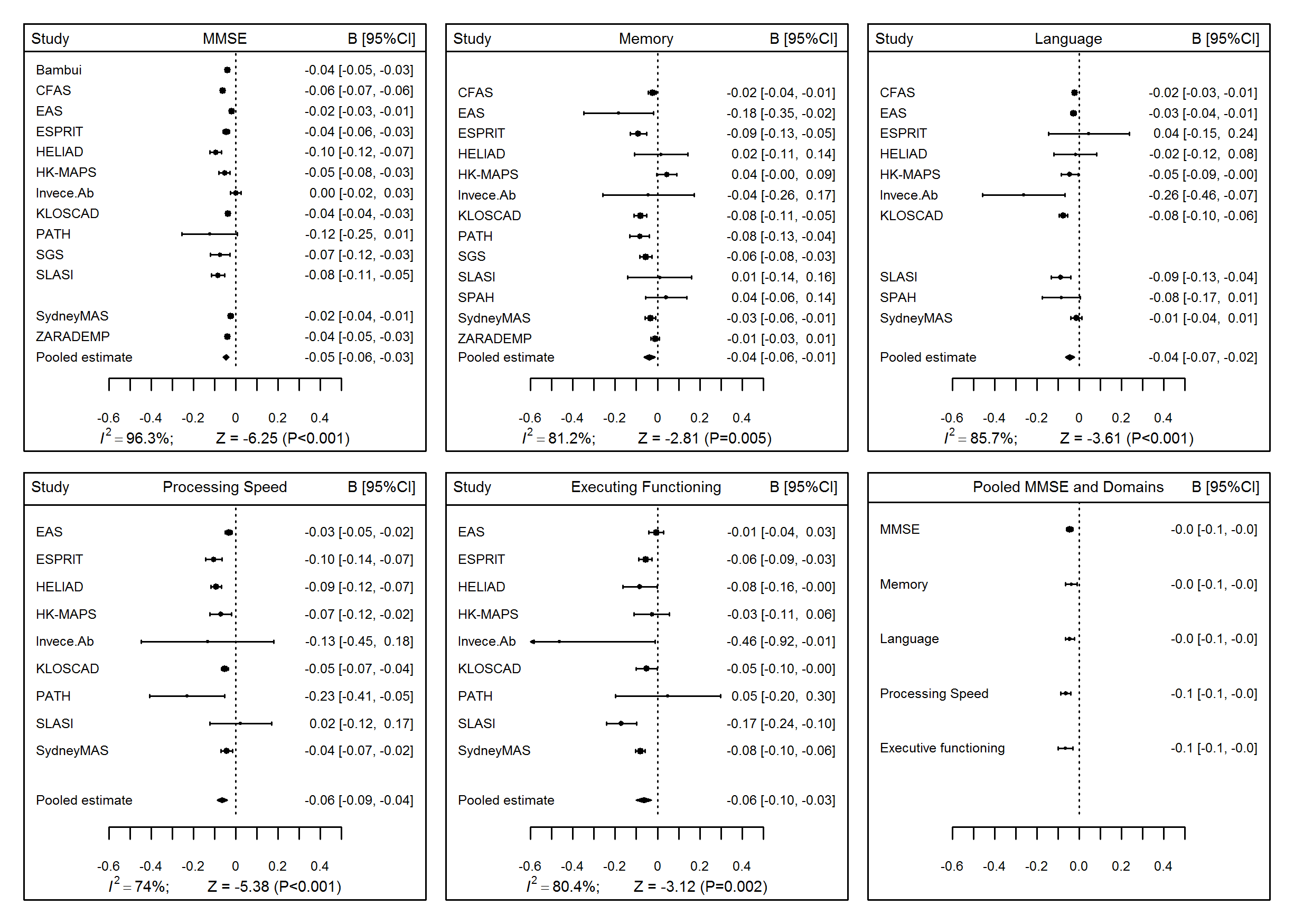

Supplement: S1 Fig — Z- and p-values are for the statistical tests of significance of the pooled values. (TIF) [file pmed.1002261.s002.tif]
