# Supplementary material for: Age-related cognitive decline and associations with sex, education and apolipoprotein E genotype across ethnocultural groups and geographic regions: a collaborative cohort study
Source: PLoS Med. 2017 Mar 21;14(3):e1002261. doi: 10.1371/journal.pmed.1002261 (PMC5360220; doi:10.1371/journal.pmed.1002261)
Supplement: S1 Table — (DOCX) [file pmed.1002261.s003.docx]

**S1 Table.** Number of assessment waves, time since baseline (yrs, mean±SD and range), and number of individuals assessed with the MMSE for baseline and each follow-up wave.*

| **Study** | **Waves**† | **Baseline no.** | **Wave 2** | **Wave 3** | **Wave 4** |
| --- | --- | --- | --- | --- | --- |
| CFAS | 3 | 12362 | 2.1±0.2 (1.8–3.5); 8447 | 9.9±0.6 (7.7–11.9); 3064 | - |
| ESPRIT | 4 | 2235 | 1.7±0.2 (0.6–2.7); 1934 | 3.8±0.2 (2.6–5.6); 1664 | 7.6±0.2 (6.8-9.0); 1244 |
| HELIAD | 2 | 1081 | 2.8±0.6 (1.2–6.8); 467 | - | - |
| HK-MAPS | 3 | 751 | 1.9±0.4 (1.2–3.4); 523 | 5.4±0.3 (4.5–6.5); 447 | - |
| Invece.Ab | 2 | 1191 | 2.2±0.2 (1.5–3.2); 1035 | - | - |
| KLOSCAD | 2 | 6739 | 2.0±0.3 (0.7–5.0); 5064 | - | - |
| PATH | 3 | 2543 | 4.1±0.2 (3.1–5.0); 2184 | 8.1±0.3 (7.2–9.0); 1917 | - |
| SGS | 2 | 2094 | 2.0±0.0 (1.9–2.2); 856 | - | - |
| SLASI | 3 | 854 | 1.7±0.5 (0.8–3.0); 575 | 3.9±0.2 (2.8–4.5); 314 | - |
| SPAH‡ | 2 | 1957 | 2.2±0.3 (1.1–4.1); 1552 | - | - |
| Sydney MAS | 4 | 1037 | 1.9±0.1 (1.4–2.6); 884 | 4.0±0.2 (3.4–4.8); 773 | 5.9±0.2 (5.7–6.8); 672 |
| ZARADEMP | 3 | 4744 | 2.2±0.2 (0.7–4.0); 3214 | 4.7±0.4 (3.1–6.7); 2390 | - |

MMSE, Mini-Mental State Examination.

* The number of individuals with MMSE data is not always the same as the number with data for other tests, and for which there may be more or less, including at baseline). Bambui and EAS each had 16 waves; detailed in separate tables.

† Includes baseline.

‡ The MMSE was not used; values are for the memory test.
