# Supplementary material for: Age-related cognitive decline and associations with sex, education and apolipoprotein E genotype across ethnocultural groups and geographic regions: a collaborative cohort study
Source: PLoS Med. 2017 Mar 21;14(3):e1002261. doi: 10.1371/journal.pmed.1002261 (PMC5360220; doi:10.1371/journal.pmed.1002261)
Supplement: S2 Table — (DOCX) [file pmed.1002261.s004.docx]

**S2 Table.** Time (y) since baseline and number of individuals assessed with the Mini-Mental State Examination for the 16 annual Bambui study assessment waves.

| **Baseline** | **Wave 2** | **3** | **4** | **5** | **6** | **7** | **8** |
| --- | --- | --- | --- | --- | --- | --- | --- |
| 1557 | 1; 1411 | 2; 1317 | 3; 1243 | 4; 1167 | 5; 1109 | 6; 1033 | 7; 1051 |
|  |  |  |  |  |  |  |  |
| **9** | **10** | **11** | **12** | **13** | **14** | **15** | **16** |
| 8; 891 | 9; 816 | 10; 724 | 11; 705 | 12; 611 | 13; 600 | 14; 444 | 15; 381 |
