# Supplementary material for: Age-related cognitive decline and associations with sex, education and apolipoprotein E genotype across ethnocultural groups and geographic regions: a collaborative cohort study
Source: PLoS Med. 2017 Mar 21;14(3):e1002261. doi: 10.1371/journal.pmed.1002261 (PMC5360220; doi:10.1371/journal.pmed.1002261)
Supplement: S3 Table — (DOCX) [file pmed.1002261.s005.docx]

**S3 Table.** Time (y, mean ± SD, and range) since baseline and number of individuals assessed with the Blessed Information Memory Concentration test* for each of the 16 EAS assessment waves.

| **Baseline** | **Wave 2** | **3** | **4** | **5** | **6** |
| --- | --- | --- | --- | --- | --- |
| 2235 | 1.2±0.5 (0.3–9.7); 1338 | 2.4±0.7 (1.6–11.4); 949 | 3.5±0.8 (2.6–11.4); 709 | 4.6±0.9 (3.6–12.4); 550 | 5.6±1.0 (4.7–5.6); 437 |
|  |  |  |  |  |  |
| **7** | **8** | **9** | **10** | **11** | **12** |
| 6.6±1.1 (5.6–16.4); 327 | 7.6±1.0 (6.6–15.5); 239 | 8.7±1.2 (7.6–16.3); 150 | 9.8±1.2 (8.8–17.4); 104 | 10.8±1.3 (9.8–19.6); 77 | 11.6±0.8 (10.8–15.5); 64 |
|  |  |  |  |  |  |
| **13** | **14** | **15** | **16** |  |  |
| 12.7±0.8 (11.8–16.6); 50 | 13.6±1.0 (12.7–17.5); 30 | 14.5±0.4 (14.1–14.9); 7 | 15.1±0.0 (15.1–15.1); 2 |  |  |

*A validated formula was used to convert scores to Mini-Mental State Examination scores.
