# Supplementary material for: Age-related cognitive decline and associations with sex, education and apolipoprotein E genotype across ethnocultural groups and geographic regions: a collaborative cohort study
Source: PLoS Med. 2017 Mar 21;14(3):e1002261. doi: 10.1371/journal.pmed.1002261 (PMC5360220; doi:10.1371/journal.pmed.1002261)
Supplement: S4 Table — (DOCX) [file pmed.1002261.s006.docx]

**S4 Table.** Ethics approvals for the individual contributing studies.

| **Study** | **Institutional Review Board** |
| --- | --- |
| Bambui | Ethics Boards of the Fundac¸a˜o Oswaldo Cruz in Rio de Janeiro and the Instituto Rene´ Rachou of the Fundac¸a˜o Oswaldo Cruz in Belo Horizonte, Brazil (14/2007 - CEPSH-CpqRR) |
| CFAS | Anglia and Oxford Multi-centre Research Ethics Committee (MREC) - 99/5/22 ; Eastern MREC – 99/5/22; Eastern MREC – 05/MREO5/37; NRES Committee East of England – 05/MRE05/37 |
| EAS | Albert Einstein College of Medicine Institutional Review Board (Approval#1996-175) |
| ESPRIT | Ethics committee (CCPPRB) of the Kremlin Bicetre hospital (n° registered 99-28) |
| HELIAD | Institutional Ethics Review Board of the University of Thessaly (ΒΕΥ846Ψ8Ν2-32Π) |
| HK-MAPS | Joint Chinese University of Hong Kong-New Territories East Cluster Clinical Research Ethics Committee (CRE-2011.101) |
| Invece.Ab | Ethics Committee of the University of Pavia (#3/2009) |
| KLOSCAD | Institutional Review Board of Seoul National University Bundang Hospital, Korea (IRB No. B-0912/089-010) |
| PATH | Australian National University Human Research Ethics Committee (#M9807, #2002/189, #2006/314, # 2010/542, #2001/2, #2009/039) |
| SPAH | Ethical Committee for the Analysis of Research Projects (CAPesq) - Hospital das Clínicas and Medical School - Project Registry Number: 257/2002; National Ethical Committee on Research (CONEP-Brazil) - Project Registry Number: 4355 |
| SGS | Institutional Review Board of the Institute of Health Science, Kyushu University (IHS-2010-22) |
| SLAS | National University of Singapore Institutional Review Board (Reference Code: 04-140) |
| Sydney MAS | University of New South Wales Human Research Ethics Committee (approval #14327) |
| ZARADEMP | Ethics committee of the Zaragoza University Hospital (CEICA # CP16/2012) |

Written consent was exclusively or predominantly obtained from participants in all studies (SPAH obtained oral consent from illiterate participants; CFAS obtained oral consent, countersigned by a witness, from participants with a physical/visual disability).
