# Supplementary material for: Age-related cognitive decline and associations with sex, education and apolipoprotein E genotype across ethnocultural groups and geographic regions: a collaborative cohort study
Source: PLoS Med. 2017 Mar 21;14(3):e1002261. doi: 10.1371/journal.pmed.1002261 (PMC5360220; doi:10.1371/journal.pmed.1002261)
Supplement: S5 Table — (DOCX) [file pmed.1002261.s007.docx]

**S5 Table.** Tests from each contributing study used to represent the cognitive domains investigated.

| Study | Memory | Language | Processing speed* | Executive functioning* |
| --- | --- | --- | --- | --- |
| Bambui | MMSE recall | - | - | - |
| CFAS | MMSE recall | Animals in 60s | - | - |
| EAS | Free and Cued Selective Reminding Test | Animals in 60s | Trail Making Test A | Trail Making Test B (if TMTA≥0 and TMTB=missing or >300 then TMTB=300s). |
| ESPRIT | MMSE recall | Animals in 30s | Trail Making Test A | Trail Making Test B |
| HELIAD | Greek Verbal Learning Test | Objects in 60s | Trail Making Test A | Trail Making Test B |
| HK-MAPS | Item “ADAS – Delayed Recall” from the Alzheimer’s Disease Assessment Scale - Cognitive | Animals in 60s | Trail Making Test A (one set each of Arabic and Chinese numbers; 120s) | Trail Making Test B (alternating Arabic and Chinese numbers; 120s) |
| Invece.Ab | Rey Auditory Verbal Learning Test, trial 7 (delay=15 min) | Mean of 4 categories (colours, animals, fruits, cities), each 120 s | Trail Making Test A | Trail Making Test B |
| KLOSCAD | Word-list Recall Test | Animals in 60s | Trail Making Test A | Trail Making Test B |
| PATH | California Verbal Learning Test (recall of first list) | - | Trail Making Test A (300s) | Trail Making Test B (300s) |
| SPAH | Item #26 (WORDDEL) from the Community Screening Interview for Dementia | Animals in 60s | - | - |
| SGS | MMSE recall | - | - | - |
| SLAS I | Rey Auditory Verbal Learning Test, trial 7 | Animals in 60s | Trail Making Test A | Trail Making Test B |
| Sydney MAS | Rey Auditory Verbal Learning Test, trial 7 | Animals in 60s | Trail Making Test A | Trail Making Test B |
| ZARADEMP | MMSE recall | - | - | - |

MMSE, Mini-Mental State Examination.

* Time limits were not imposed except where indicated.
