# Supplementary material for: Age-related cognitive decline and associations with sex, education and apolipoprotein E genotype across ethnocultural groups and geographic regions: a collaborative cohort study
Source: PLoS Med. 2017 Mar 21;14(3):e1002261. doi: 10.1371/journal.pmed.1002261 (PMC5360220; doi:10.1371/journal.pmed.1002261)
Supplement: S6 Table — (DOCX) [file pmed.1002261.s008.docx]

| **S6 Table.** Numbers and percentages of missing and deleted test scores. | | | | | | | | |
| --- | --- | --- | --- | --- | --- | --- | --- | --- |
| **Domain** | **Study** | **N** | **Missing** | | **Outliers** | | **Spikes** | |
|  |  |  | **No.** | **%** | **No.** | **%** | **No.** | **%** |
| **MMSE** | Bambui | 25696 | 10564 | 41.1 |  |  |  |  |
|  | CFAS | 39012 | 14831 | 38.0 |  |  |  |  |
|  | EAS | 36080 | 28790 | 79.8 |  |  |  |  |
|  | ESPRIT | 9036 | 1956 | 21.6 |  |  |  |  |
|  | HELIAD | 2516 | 954 | 37.9 |  |  |  |  |
|  | HK-MAPS | 2370 | 646 | 27.3 |  |  |  |  |
|  | Invece.Ab | 2642 | 416 | 15.7 |  |  |  |  |
|  | KLOSCAD | 13666 | 1859 | 13.6 |  |  |  |  |
|  | PATH | 7653 | 1003 | 13.1 |  |  |  |  |
|  | SGS | 4412 | 1373 | 31.1 |  |  |  |  |
|  | SLASI | 8412 | 2757 | 32.8 |  |  |  |  |
|  | Sydney MAS | 4148 | 782 | 18.9 |  |  |  |  |
|  | ZARADEMP | 14409 | 3966 | 27.5 |  |  |  |  |
| **Memory** | CFAS | 39012 | 14594 | 37.4 |  |  |  |  |
|  | ESPRIT | 9036 | 1958 | 21.7 |  |  |  |  |
|  | SGS | 4412 | 1359 | 30.8 |  |  |  |  |
|  | ZARADEMP | 14409 | 4098 | 28.4 |  |  |  |  |
|  | EAS | 36080 | 35034 | 97.1 |  |  |  |  |
|  | HELIAD | 2516 | 923 | 36.7 |  |  | 121 | 7.6 |
|  | HK-MAPS | 2370 | 987 | 41.6 |  |  | 144 | 10.4 |
|  | Invece.Ab | 2642 | 664 | 25.1 |  |  |  |  |
|  | KLOSCAD | 13666 | 2036 | 14.9 |  |  |  |  |
|  | PATH | 7653 | 974 | 12.7 |  |  |  |  |
|  | SPAH | 4144 | 411 | 9.9 |  |  |  |  |
|  | SLASI | 5608 | 4637 | 82.7 |  |  | 43 | 4.4 |
|  | Sydney MAS | 4148 | 948 | 22.9 |  |  | 143 | 4.5 |
| **Language** | CFAS | 39012 | 15275 | 39.2 |  |  |  |  |
|  | EAS | 36080 | 28919 | 80.2 |  |  |  |  |
|  | ESPRIT | 9036 | 7788 | 86.2 | 4 | 0.3 |  |  |
|  | HK-MAPS | 2370 | 635 | 26.8 | 7 | 0.4 |  |  |
|  | KLOSCAD | 13666 | 1991 | 14.6 |  |  |  |  |
|  | SPAH | 4144 | 411 | 9.9 | 3 | 0.1 | 103 | 2.8 |
|  | SLASI | 5608 | 4633 | 82.6 | 4 | 0.4 |  |  |
|  | Sydney MAS | 4148 | 822 | 19.8 |  |  |  |  |
|  | HELIAD | 2516 | 847 | 33.7 |  |  |  |  |
|  | Invece.Ab | 2642 | 453 | 17.1 |  |  |  |  |
| **Processing Speed** | EAS | 36080 | 29234 | 81.0 | 19 | 0.3 |  |  |
|  | ESPRIT | 9036 | 3928 | 43.5 | 28 | 0.5 |  |  |
|  | HELIAD | 2516 | 1006 | 40.0 | 5 | 0.3 |  |  |
|  | Invece.Ab | 2642 | 481 | 18.2 | 12 | 0.6 |  |  |
|  | SLASI | 5608 | 5064 | 90.3 | 10 | 1.8 |  |  |
|  | Sydney MAS | 4148 | 932 | 22.5 | 11 | 0.3 |  |  |
|  | HK-MAPS | 2370 | 1057 | 44.6 |  |  |  |  |
|  | KLOSCAD | 13666 | 2195 | 16.1 |  |  | 553 | 4.8 |
|  | PATH | 7653 | 3567 | 46.6 | 8 | 0.2 |  |  |
| **Executive Functioning** | EAS | 36080 | 29232 | 81.0 |  |  | 832 | 12.1 |
|  | ESPRIT | 9036 | 4110 | 45.5 | 15 | 0.3 |  |  |
|  | HELIAD | 2516 | 1604 | 63.8 | 4 | 0.4 |  |  |
|  | Invece.Ab | 2642 | 800 | 30.3 | 4 | 0.2 |  |  |
|  | SLASI | 5608 | 5192 | 92.6 | 3 | 0.7 |  |  |
|  | Sydney MAS | 4148 | 1139 | 27.5 | 14 | 0.5 |  |  |
|  | HK-MAPS | 2370 | 1205 | 50.8 |  |  | 421 | 36.1 |
|  | KLOSCAD | 13666 | 2314 | 16.9 |  |  | 4014 | 35.4 |
|  | PATH | 7653 | 3612 | 47.2 | 15 | 0.4 |  |  |

N is the total number of possible scores if all individuals at baseline provided data for all assessment waves. Missing indicates no data in the original study file. Outliers were scores >3 SDs from the mean. Spikes refer to scores deleted to obtain distributions that gave adequate model fit or convergence.
