# Supplementary material for: Age-related cognitive decline and associations with sex, education and apolipoprotein E genotype across ethnocultural groups and geographic regions: a collaborative cohort study
Source: PLoS Med. 2017 Mar 21;14(3):e1002261. doi: 10.1371/journal.pmed.1002261 (PMC5360220; doi:10.1371/journal.pmed.1002261)
Supplement: S10 Table — (DOCX) [file pmed.1002261.s012.docx]

| **S10 Table.** For predominantly white studies, meta-analyses of the fixed effects of age^2^ on MMSE and cognitive domain scores. | | | | | | |
| --- | --- | --- | --- | --- | --- | --- |
| **Study** | **MMSE** | **Memory** | **Language** | **Proc Speed** | | **Executive Fn** |
| Bambui |  |  |  |  | |  |
| CFAS | -0.06 (0.00) | -0.02 (0.01) | -0.02 (0.00) |  | |  |
| EAS |  |  |  |  | |  |
| ESPRIT | -0.04 (0.01) | -0.09 (0.02) | 0.04 (0.10) | -0.10 (0.02) | | -0.06 (0.02) |
| HELIAD | -0.10 (0.01) | 0.02 (0.06) | -0.02 (0.05) | -0.09 (0.01) | | -0.08 (0.04) |
| HK-MAPS |  |  |  |  | |  |
| Invece.Ab | 0.00 (0.01) | -0.04 (0.11) | -0.26 (0.10) | -0.13 (0.16) | | -0.46 (0.23) |
| KLOSCAD |  |  |  |  | |  |
| PATH | -0.12 (0.07) | -0.08 (0.02) |  | -0.23 (0.09) | | 0.05 (0.13) |
| SPAH |  |  |  |  | |  |
| SGS |  |  |  |  | |  |
| SLASI |  |  |  |  | |  |
| Sydney MAS | -0.02 (0.01) | -0.03 (0.01) | -0.01 (0.01) | -0.04 (0.01) | | -0.08 (0.01) |
| ZARADEMP | -0.04 (0.00) | -0.01 (0.01) |  |  | |  |
|  |  |  |  |  | |  |
| Pooled across studies (random effects) | -0.05 (0.01) p=<0.001 | -0.04 (0.01) p<0.001 | -0.03 (0.04) p=0.329 | -0.09 (0.02) p<0.001 | | -0.07 (0.04) p=0.041 |
| I^2^ | 96.9% | 52.3% | 92.0% | 66.1% | | 85.4% |
|  |  |  |  |  | |  |
| Pooled across studies (random effects) No baseline dementia | -0.05 (0.01) p<0.001 | -0.04 (0.01) p<0.001 | -0.05 (0.08) p=0.487 | -0.08 (0.01) p<0.001 | | -0.08 (0.05) p=0.092 |
| I^2^ | 93.1% | 42.2% | 98.5% | 52.1% | | 91.9% |
| MMSE, Mini-Mental State Examination.  Values are presented as regression coefficient (B), with standard error in parentheses, and with age in units of decades.  The last two rows are for the analyses repeated with cases of dementia at baseline removed. | | | | |  |  |
