# Supplementary material for: Age-related cognitive decline and associations with sex, education and apolipoprotein E genotype across ethnocultural groups and geographic regions: a collaborative cohort study
Source: PLoS Med. 2017 Mar 21;14(3):e1002261. doi: 10.1371/journal.pmed.1002261 (PMC5360220; doi:10.1371/journal.pmed.1002261)
Supplement: S13 Table — (DOCX) [file pmed.1002261.s015.docx]

| **S13 Table.** Meta-analyses of the fixed effects of sex on MMSE and cognitive domain scores. | | | | | | |
| --- | --- | --- | --- | --- | --- | --- |
| **Study** | **MMSE** | **Memory** | **Language** | **Proc Speed** | | **Executive Fn** |
| Bambui | 0.0090 (0.0090) |  |  |  | |  |
| CFAS | 0.0300 (0.0021) | 0.0147 (0.0111) | 0.0585 (0.0066) |  | |  |
| EAS | 0.0156 (0.0039) | 0.0101 (0.0835) | 0.0215 (0.0101) | -0.0224 (0.0103) | | 0.0233 (0.0219) |
| ESPRIT | 0.0021 (0.0060) | -0.1815 (0.0217) | 0.1563 (0.0825) | -0.0110 (0.0170) | | -0.0232 (0.0157) |
| HELIAD | 0.0290 (0.0096) | -0.5717 (0.0623) | -0.1717 (0.0518) | 0.0776 (0.0138) | | 0.0889 (0.0321) |
| HK-MAPS | 0.1124 (0.0187) | -0.0233 (0.0348) | 0.0067 (0.0340) | 0.2858 (0.0375) | | 0.0985 (0.0553) |
| Invece.Ab | -0.0017 (0.0019) | -0.0807 (0.0156) | -0.0002 (0.0183) | 0.0247 (0.0203) | | 0.0151 (0.0247) |
| KLOSCAD | 0.0057 (0.0035) | -0.2053 (0.0247) | 0.0407 (0.0163) | 0.0505 (0.0103) | | 0.0989 (0.0308) |
| PATH | -0.0589 (0.0379) | -0.1879 (0.0199) |  | -0.1238 (0.0327) | | 0.0002 (0.0427) |
| SPAH |  | -0.2489 (0.0565) | 0.1059 (0.0536) |  | |  |
| SGS | -0.0580 (0.0198) | -0.0901 (0.0122) |  |  | |  |
| SLASI | 0.0501 (0.0333) | -0.5169 (0.1303) | 0.0653 (0.0493) | 0.3419 (0.1287) | | 0.0344 (0.0627) |
| Sydney MAS | -0.0062 (0.0036) | -0.1480 (0.0187) | -0.0242 (0.0182) | -0.0061 (0.0130) | | -0.0053 (0.0099) |
| ZARADEMP | 0.0540 (0.0058) | -0.0609 (0.0179) |  |  | |  |
|  |  |  |  |  | |  |
| Pooled across studies (random effects) | 0.0155 (0.0106) p=0.144 | -0.1634 (0.0448) p<0.001 | 0.0199 (0.0191) p=0.296 | 0.0505 (0.0417) p=0.225 | | 0.0284 (0.0161) p=0.078 |
| I^2^ | 98.6% | 98.2% | 89.5% | 98.2% | | 72.5% |
|  |  |  |  |  | |  |
| Pooled across studies (random effects) No baseline dementia | 0.0170 (0.0134) p=0.202 | -0.1727 (0.0472) p<0.001 | 0.0213 (0.0178) p=0.233 | 0.0445 (0.0376) p=0.236 | | 0.0286 (0.0143) p=0.045 |
| I^2^ | 98.7% | 98.3% | 87.3% | 98.1% | | 36.2% |
| MMSE, Mini-Mental State Examination.  Values are presented as regression coefficient (B), with standard error in parentheses.  Sex is coded as female = 0 and male = 1.  The last two rows are for the analyses repeated with cases of dementia at baseline removed. | | | | |  |  |
