# Supplementary material for: Age-related cognitive decline and associations with sex, education and apolipoprotein E genotype across ethnocultural groups and geographic regions: a collaborative cohort study
Source: PLoS Med. 2017 Mar 21;14(3):e1002261. doi: 10.1371/journal.pmed.1002261 (PMC5360220; doi:10.1371/journal.pmed.1002261)
Supplement: S14 Table — (DOCX) [file pmed.1002261.s016.docx]

| **S14 Table.** Meta-analyses of sex by age interactions on MMSE and cognitive domain scores. | | | | | | |
| --- | --- | --- | --- | --- | --- | --- |
| **Study** | **MMSE** | **Memory** | **Language** | **Proc Speed** | | **Executive Fn** |
| Bambui | 0.050 (0.008) |  |  |  | |  |
| CFAS | 0.036 (0.003) | 0.032 (0.016) | 0.004 (0.008) |  | |  |
| EAS | 0.019 (0.006) | -0.005 (0.108) | -0.002 (0.011) | 0.020 (0.012) | | 0.006 (0.026) |
| ESPRIT | 0.009 (0.012) | -0.065 (0.034) | -0.207 (0.129) | 0.057 (0.030) | | -0.063 (0.025) |
| HELIAD | 0.019 (0.021) | 0.012 (0.110) | 0.048 (0.091) | 0.048 (0.019) | | 0.158 (0.067) |
| HK-MAPS | 0.045 (0.027) | 0.071 (0.046) | 0.037 (0.043) | 0.069 (0.045) | | 0.113 (0.075) |
| Invece.Ab | 0.001 (0.005) | 0.089 (0.053) | 0.090 (0.051) | -0.101 (0.066) | | -0.063 (0.094) |
| KLOSCAD | 0.010 (0.004) | 0.055 (0.030) | 0.036 (0.020) | 0.037 (0.014) | | -0.004 (0.039) |
| PATH | -0.015 (0.045) | -0.005 (0.018) |  | -0.210 (0.048) | | 0.019 (0.064) |
| SPAH |  | 0.100 (0.086) | -0.021 (0.079) |  | |  |
| SGS | 0.027 (0.034) | -0.080 (0.021) |  |  | |  |
| SLASI | 0.026 (0.027) | -0.200 (0.112) | 0.095 (0.043) | -0.062 (0.141) | | 0.041 (0.053) |
| Sydney MAS | 0.018 (0.007) | -0.020 (0.020) | -0.013 (0.020) | -0.033 (0.019) | | 0.018 (0.014) |
| ZARADEMP | 0.037 (0.007) | 0.028 (0.019) |  |  | |  |
|  |  |  |  |  | |  |
| Pooled across studies (random effects) | 0.023 (0.006) p<0.001 | 0.004 (0.017) p=0.810 | 0.014 (0.011) p=0.202 | -0.007 (0.029) p=0.795 | | 0.012 (0.019) p=0.542 |
| I^2^ | 84.5% | 72.6% | 44.6% | 91.7% | | 55.2% |
|  |  |  |  |  | |  |
| Pooled across studies (random effects) No baseline dementia | 0.016 (0.009) p=0.089 | 0.005 (0.010) p=0.577 | 0.012 (0.011) p=0.267 | -0.001 (0.033) p=0.982 | | -0.013 (0.024) p=0.584 |
| I^2^ | 91.7% | 20.2% | 43.6% | 94.4% | | 68.8% |
| MMSE, Mini-Mental State Examination.  Values are presented as regression coefficient (B), with standard error in parentheses, and with age in units of decades.  Sex is coded as female = 0 and male = 1.  The last two rows are for the analyses repeated with cases of dementia at baseline removed. | | | | |  |  |
