# Supplementary material for: Age-related cognitive decline and associations with sex, education and apolipoprotein E genotype across ethnocultural groups and geographic regions: a collaborative cohort study
Source: PLoS Med. 2017 Mar 21;14(3):e1002261. doi: 10.1371/journal.pmed.1002261 (PMC5360220; doi:10.1371/journal.pmed.1002261)
Supplement: S1 Text — (DOCX) [file pmed.1002261.s021.docx]

**COSMIC ANALYSIS PLAN FOR PAPER ON LONGITUDINAL RELATIONSHIPS OF AGE, SEX, EDUCATION AND APOE E4 CARRIER STATUS ON COGNITION**

**RESEARCH QUESTIONS**

- What is the longitudinal relationship between age, and a number of mental tests representing global cognitive ability and separate cognitive domains.
- In particular, as well as an expected decline with age, is there a significant increasing rate of decline with increasing age?
- What is the effect of sex, years of education and APOE ε4 carrier status on levels of cognitive performance and rates of decline in each of these cognitive measures?

**STATISTICAL ANAYSIS**

**Overview of Procedure:**

After selection of tests and removal of outliers, obtain standardized scores (as Z-scores) for each of the tests based on internal norms. With standardized scores as DVs, use (generalized) linear mixed models (G)LMMs to evaluate, within each study separately, the fixed effects of age, age-squared, sex, education and APOE ε4 status, as well as the interaction of the last 3 variables with age and age-squared. Then use meta-analysis to obtain pooled values of the fixed effects of each of the variables in the equation.

**Steps in Statistical Analyses:**

1. ***Selection of tests:*** Tests will be selected to represent individual domains, seeking to maximise the similarity of type of test within each domain across studies, to minimise the amount of missing study/test combinations, and so that the tests are widely recognised as markers of the domains they are chosen to represent.
2. ***Removal of outliers:*** Inspect the distribution of test scores for any anomalies or unexpected patterns. Delete scores greater than 3 standard deviations from the mean. For skewed variables, transform using an appropriate function to reduce skewness as much as possible, and use the mean and SD of the transformed variable to select outliers according to the above rule.
3. ***Calculation of standardized scores:*** For each test/study combination, use linear mixed models to estimate the mean and SD at 75 years of age, and with years of education =9, and sex = 0.5. Note that the means and SD are to be estimated at common values across the studies, to make the standardized scores more comparable between studies, despite variation in the distributions of these variables from study to study.
4. ***Estimating fixed effects of variables within each study:*** With standardized scores as the DVs, use either LMM or GLMM to estimate the fixed effects of age, age-squared, sex, education and APOE ε4 status, as well as the interaction of the last 3 variables with age and age-squared. Before analyses, centre age to 75 years (the approximate mean value across all studies and waves) in order to minimise multicollinearity between age and other terms in the equation (age-squared and interactions with age). Include in the models random effects for the intercept, age, and age-squared. The GLMM is to be used for non-normally distributions of the standardized scores, and different distributions and link functions available in the R program will be examined to produce the best model fit.
5. ***Use meta-analysis to obtain pooled values of fixed effects:*** The estimated values of the fixed effects of the variables in the mixed models, together with their standard errors of measurement, will be entered into the meta-analysis program, using the random effects model, and inverse variance method for the weighting of studies. As well obtaining the pooled estimates and their SEMs, obtain the I-squared measure of heterogeneity as well, for entry into tables and/or forest plots for reporting the results.
6. ***Examination of pooled values separately from studies with predominantly Asian or white participants:*** The above meta-analysis will be used on these two groups of studies, separately, to obtain separate pooled estimates of the fixed effects. The SEMs given in the output of the meta-analysis program will then be used to examine the statistical significance of the difference between pooled values of each of the cognitive measures, derived from the two groups of studies. This will be done by calculating the SEM of the difference between the two pooled values, equal to the square-root of the sum of squares of the two SEMs. If the difference between the two pooled values exceeds 1.96 the value of this SEM of the difference, then that will indicate that the difference in the pooled values is significant at the .05 level.

**VARIATIONS FROM PLANNED STATISTICAL ANALYSES**

1. When examining the mixed models, it was found that the inclusion of random effects for the age-squared term did not improve model fit, so random effects were only used for the intercept and age variables.
2. When inspecting the distributions of the measures of cognition we observed some “spikes”. The most prominent of these were for timed tests where the test was terminated after a predetermined period, and a score equal to that time was recorded as the score. It was found that distributions containing spikes made it difficult to fit the distribution using GLMM, so scores contributing to spikes were deleted. However, to examine whether the fixed effects obtained with these scores deleted affected our results, the meta-analyses were repeated with the studies featuring spikes removed.
